# Lagos Bat Virus, South Africa

**DOI:** 10.3201/eid1203.051306

**Published:** 2006-03

**Authors:** Wanda Markotter, Jenny Randles, Charles E. Rupprecht, Claude T. Sabeta, Peter J. Taylor, Alex I. Wandeler, Louis H. Nel

**Affiliations:** * University of Pretoria, Pretoria, South Africa;; † Allerton Veterinary Laboratory, Pietermaritzburg, South Africa;; ‡ Centers for Disease Control and Prevention, Atlanta, Georgia, USA;; § Onderstepoort Veterinary Research Institute, Pretoria, South Africa;; ¶ Natural Science Museum, Durban, South Africa;; # Canadian Food Inspection Agency, Nepean, Ontario, Canada

**Keywords:** Lagos bat virus, rabies, rabies-related viruses, lyssaviruses, nucleoprotein, bats, Megachiroptera, Africa, South Africa

## Abstract

Three more isolates of Lagos bat virus were recently recovered from fruit bats in South Africa after an apparent absence of this virus for 13 years. The sporadic occurrence of cases is likely due to inadequate surveillance programs for lyssavirus infections among bat populations in Africa.

Since 2003, we have embarked on a passive surveillance study to collect and identify bats with neurologic disease signs that may indicate encephalitis due to lyssavirus infection. Consequently, 3 new cases of Lagos bat virus (LBV) infection in fruit bats were identified in South Africa, 1 each in 2003, 2004, and 2005. LBV is a member of the *Lyssavirus* genus in the *Rhabdoviridae* family. Rabies virus (RABV) was first isolated as a unique virus within this group. However, after the isolation of rabies-related viruses in Africa and Europe in the mid-1950s, the *Lyssavirus* genus was created, and rabies virus (genotype 1) was designated as the type-species member of the genus. At least 7 different major *Lyssavirus* species (genotypes) are recognized (*1*), but the genus will be expanded to include organisms isolated from Eurasia in recent years (*2*). At present, 4 *Lyssavirus* species (genotypes) are recognized in Africa. Of these, RABV (genotype 1) occurs worldwide, but LBV (genotype 2), Mokola virus (genotype 3), and Duvenhage virus (genotype 4) have not been encountered outside of Africa. Although RABV infection of bats is well known in the Americas, this virus has only been associated with infections of terrestrial mammals on the African continent. Mokola virus has also been isolated only from various terrestrial species, never bats (*3*). Both LBV and Duvenhage virus are thought to be bat viruses, although LBV infections of terrestrial animals have been reported (*4**,**5*). RABV is a zoonotic agent throughout Africa; Duvenhage virus and Mokola virus, but not LBV, have also been responsible for rare zoonotic events (*3**,**6*).

LBV was first isolated from a fruit bat in 1956 in Nigeria (*7*), but not until 1970 was it identified as a rabies-related virus (*8*). Since then (and before this report), 11 more isolations of LBV were made throughout Africa (Table 1), including 5 isolates from South Africa.

**Table 1:** Lagos bat virus isolates recorded to date

Geographic origin	Year of isolation	Animal	Reference
Lagos, Nigeria	1956	Bat (*Eidolon helvum*)	(*7*)
Bozo, Central African Republic	1974	Bat (*Micropterus pusillus*)	(*9*)
Pinetown, South Africa (3 isolates)	1980	Bat (*Epomophorus wahlbergi*)	(*10**,**11*)
Stanger, South Africa	1982	Cat	(*11*)
Kindia, Senegal	1985	Bat (*Nycteris cambiensis*)	(*12*)
Dakar, Senegal	1985	Bat (*E. helvum*)	(*12*)
Dorowa, Zimbabwe	1986	Cat	(*4*)
Durban, South Africa	1990	Bat (*E. wahlbergi*)	(*6*)
Ethiopia	Before 1992	Dog	(*5*)
Egypt	1999	Bat (*Roussetus aegyptiacus*)	(*13*)
Durban, South Africa	2003	Bat (*E. wahlbergi*)	This report
Durban, South Africa	2004	Bat (*E. wahlbergi*)	This report
Durban, South Africa	2005	Bat (*E. wahlbergi*)	This report

## The Case

In June 2003, an *Epomophorus wahlbergi* carcass was recovered in Durban, KwaZulu-Natal, after the bat was caught by a domestic cat. In August 2004, a resident of Umbilo, Durban, found a dead *E. wahlbergi* fruit bat on her lawn one morning after hearing squeaking noises around the house during the night. The fluorescent-antibody test (FAT), performed on brain material, was positive for lyssavirus antigens, and virus was isolated in both cases when suckling mice died 9–14 days after intracerebral injection with brain suspensions. Antigenic typing was carried out with a panel of anti-lyssavirus nucleocapsid monoclonal antibodies (prepared by the Centre of Expertise for Rabies, Canadian Food Inspection Agency, Nepean, Ontario, Canada). These analyses identified both new isolates as LBV (genotype 2) (Table 2). Additional characterization was accomplished by polymerase chain reaction (PCR) and sequencing of a 457-bp region of the nucleoprotein-encoding gene with a novel set of PCR and sequencing primers specific for LBV (LagNF (5´-GGGCAGATATGACGCGAGA-3´) and LagNR (5´-TTGACCGGGTTCAAACATC-3´). Briefly, total RNA was extracted from infected tissue by using TRIzol (Invitrogen, Croningen, the Netherlands) according to the manufacturer's instructions. Complementary DNA was produced by a reverse transcription reaction (RT) and used in subsequent PCR. PCR products were purified by using the Wizard SV PCR and Gel purification kit (Promega, Madison, WI, USA). The purified products were then sequenced by using the Big Dye Termination Cycle Sequencing Ready Reaction Kit 3.1 (Applied Biosystems, Foster City, CA, USA), according to the manufacturer's protocol, with subsequent analysis on an Applied Biosystems 377 DNA automated sequencer.

**Table 2:** Immunofluorescence reaction of a panel of 16 monoclonal antibodies against the nucleoprotein of Lagos bat virus isolations, South Africa, 2003 and 2004*

Antibody	Canid biotype (GT1)	Mongoose biotype (GT1)	Lagos bat virus (GT2)	Mokola virus (GT3)	Duvenhage virus (GT4)	2003 isolate	2004 isolate
1C5	–	–	–	–	–	–	–
26AB7	+++	Variable	–	–	–	–	–
26BE2	+++	Variable	–	–	–	–	–
32GD12	Variable	Variable	–	–	–	–	–
38HF2	+++	+++	+++	+++	+++	+++	+++
M612	–	–	+++	–	–	+++	+++
M837	–	–	–	–	+++	–	–
M850	–	Variable	–	–	+++	–	–
M853	+++	–	–	–	+++	–	–
M1001	–	–	–	+++	–	–	–
M1335	–	Variable	–	Variable	–	–	–
M1386	–	+++	–	–	–	–	–
M1400	–	Variable	–	–	–	–	–
M1407	++	Variable	–	–	–	–	–
M1412	++	Variable	–	–	–	–	–
M1494	–	Variable	–	–	+++	–	–

*Typical immunofluorescence antibody pattern observed for all lyssavirus genotypes present on the African continent (genotype [GT] 1, 2, 3, and 4) are also included as a reference: –, no specific fluorescence; ++, strong fluorescence; +++, very strong fluorescence.

In June 2005, a caretaker/gardener at a communal outdoor sports complex in the Bluff, Durban, found a bat on the lawns of the complex. At the time, birds were picking at it, and on closer inspection, it was found to be an immobile adult animal with a pup attached to it. The caretaker collected both bats and placed them in a nearby tree. Later, the bats, still attached to each other, were again found on the ground, where eyewitnesses also saw a cat toying with it. The animals were then presented to a local bat rehabilitator. The adult animal died and was submitted for diagnostic testing, but results of FAT carried out on brain smears were repeatedly negative. The pup had at least 1 evident bite wound, presumably from the cat, but otherwise appeared healthy and was cared for by the rehabilitator. Although the pup was reported to be feeding and doing well, it suddenly died ≈4 days after being found, on June 21, 2005. By this time, RT-PCR and nucleotide sequencing assays, carried out as described above, showed LBV in brain material from the adult. Antigenic typing was not performed because the level of lyssavirus antigen in the brain matter was undetectable. In the meantime, the carcass of the pup was recovered, and brain material was subjected to FAT and diagnostic RT-PCR. Although the RT-PCR results were inconclusive, the FAT results were negative.

DNA sequencing information from each case was compared with nucleoprotein sequence information for LBV and other lyssavirus species (genotypes) available in the public domain (GenBank). ClustalW was used to produce sequence alignments and generate a phylogenetic tree (Figure). A graphic representation of the trees was constructed with the TreeView program. In this phylogeny, the 3 new LBV isolates segregate together with previously identified LBV isolates from Ethiopia (AY333110) (*7*) and Nigeria (U22842) (*5*). The recent isolates from South Africa share a close sequence homology with the isolate from Ethiopia. This finding warrants further investigation.

**Figure:**
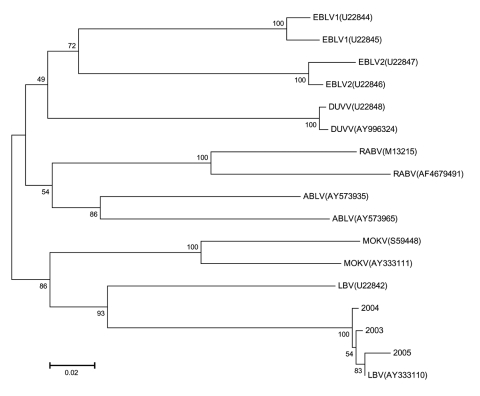
A neighbor-joining tree comparing 457 nucleotides of the nucleoprotein-encoding genes of the new Lagos bat isolations made in South Africa (bat 2003 [DQ201178], 2004 [DQ201179], and 2005 [DQ201180]) with representative sequences of the 7 genotypes of lyssaviruses obtained from GenBank. GenBank accession numbers are indicated on the figure. The bootstrap values were determined with 1,000 replicates.

## Conclusions

Although LBV is rare and has not been reported in South Africa in 13 years, a small-scale passive surveillance effort in KwaZulu-Natal, South Africa, enabled us to identify 3 new isolations of LBV in a relatively short time. This finding reemphasizes our lack of understanding of the true prevalence of lyssaviruses in Africa because of poor surveillance for non-rabies viruses (and, in fact, RABV) throughout the continent. Human infections with LBV have not been documented to date; however, this virus has been reported in domestic animals (2 cats [4] and a dog [5]). We describe close contact between humans and other animals and LBV-infected bats. Cross-neutralization data obtained in rodent models show that rabies preexposure and postexposure prophylaxis is unlikely to be effective against LBV (*14*). We have shown that LBV infection may be present in bat populations; consequently, we recommend appropriate precautions and use of proper personal protection equipment, such as gloves, when interacting with these animals. Even though the value of rabies vaccination is doubtful, it should be considered in light of the potential for cross-reactivity (*15*) and the lack of alternatives. Surveillance should be maintained as part of a strategy to better understand the epidemiology of LBV. Cumulatively, all available evidence indicates that LBV is likely persistently maintained in Megachiroptera populations in South Africa and other African countries where LBV has been reported in the past.
